# (*E*)-4-Methyl-*N*-[(5-nitro­thio­phen-2-yl)methyl­idene]aniline

**DOI:** 10.1107/S1600536812026062

**Published:** 2012-06-16

**Authors:** Ümit Ceylan, Sümeyye Gümüş, Erbil Ağar, Mustafa Serkan Soylu

**Affiliations:** a Department of Physics, Faculty of Arts and Sciences, Ondokuz Mayıs University, 55139 Samsun, Turkey; b Department of Chemistry, Faculty of Arts and Sciences, Ondokuz Mayıs University, 55139 Samsun, Turkey; c Department of Physics, Faculty of Arts and Sciences, Giresun University, 28100 Giresun, Turkey

## Abstract

In the crystal structure of the title compound, C_12_H_10_N_2_O_2_S, the benzene and the 2-nitro­thio­phene rings make a dihedral angle of 7.47 (12)°. The dihedral angle between the nitro group and the attached ring is 1.9 (6)°.

## Related literature

For related structures, see: Demirtaş *et al.* (2009 ▶); Ceylan *et al.* (2011 ▶).
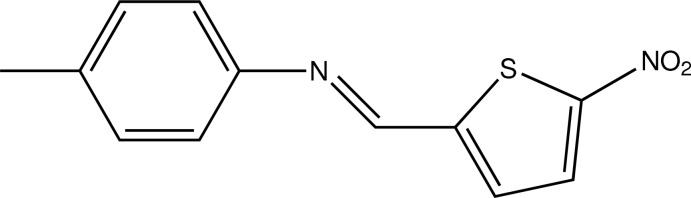


## Experimental

### 

#### Crystal data

C_12_H_10_N_2_O_2_S
*M* *_r_* = 246.28
Monoclinic, 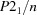

*a* = 4.7661 (4) Å
*b* = 22.8201 (18) Å
*c* = 10.7793 (7) Å
β = 92.704 (7)°
*V* = 1171.08 (15) Å^3^
*Z* = 4
Mo *K*α radiation
μ = 0.26 mm^−1^
*T* = 296 K
0.17 × 0.15 × 0.12 mm

#### Data collection

Oxford Diffraction SuperNova Eos diffractometer
Absorption correction: multi-scan (*CrysAlis PRO*; Oxford Diffraction, 2009 ▶) *T*_min_ = 0.771, *T*_max_ = 1.000
3997 measured reflections
2211 independent reflections
1549 reflections with *I* > 2σ(*I*)
*R*_int_ = 0.064

#### Refinement

*R*[*F* ^2^ > 2σ(*F* ^2^)] = 0.063
*wR*(*F* ^2^) = 0.177
*S* = 1.05
2211 reflections
155 parameters
H-atom parameters constrained
Δρ_max_ = 0.26 e Å^−3^
Δρ_min_ = −0.28 e Å^−3^

### 

Data collection: *CrysAlis PRO* (Oxford Diffraction, 2009 ▶); cell refinement: *CrysAlis PRO*; data reduction: *CrysAlis PRO*; program(s) used to solve structure: *WinGX* (Farrugia, 1997 ▶) and *SHELXS97* (Sheldrick, 2008 ▶); program(s) used to refine structure: *SHELXL97* (Sheldrick, 2008 ▶); molecular graphics: *OLEX2* (Dolomanov *et al.* 2009 ▶) and *ORTEP-3 for Windows* (Farrugia, 1997 ▶);; software used to prepare material for publication: OLEX2 (Dolomanov *et al.* 2009 ▶), *WinGX* (Farrugia, 1999 ▶) and *PLATON* (Spek, 2009 ▶).

## Supplementary Material

Crystal structure: contains datablock(s) I, New_Global_Publ_Block. DOI: 10.1107/S1600536812026062/nc2283sup1.cif

Structure factors: contains datablock(s) I. DOI: 10.1107/S1600536812026062/nc2283Isup2.hkl

Additional supplementary materials:  crystallographic information; 3D view; checkCIF report

## Figures and Tables

**Fig. 1.**
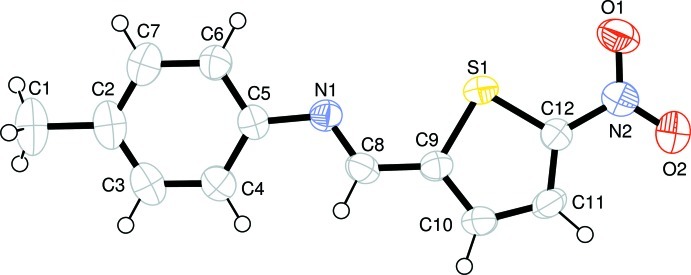
The asymmetric unit of the title compound, showing the atomic numbering scheme. Displacement ellipsoids are drawn at the 50% probability level.

## supplementary crystallographic information

### Comment

The title compound was by the reaction between 5-nitrothiophene-2-carboxaldehyde and *p*-Toluidine. For the identification of this compound a single crystal structure analysis was performed. It is noted that some 2-nitrothiophene structures have already been reported in literature (Demirtaş *et al.* 2009; Ceylan *et al.* 2011). In the crystal structure the dihedral angle between the 6-membered and the 2-nitrothiophene rings amount to 7.47 (12)° and the torsion angle along C5—N1—C8—C9 is 179.7 (3)°. Both rings are in a trans arrangement with respect to the C-N double bond and the nitro group is oriented almost coplanar to the 2-nitrothiophene plane (Fig. 1).

### Experimental

A mixture of 5-nitrothiophene-2-carboxaldehyde (0.011 g 0.066 mmol) in 20 ml e thanol and of *p*-Toluidine (0.007 g 0.066 mmol) in 20 ml ethanol was refluxed for 1 h. Single crystals suitable for X-ray analysis were obtained from a solution of the title compound in ethanol by slow evaporation of the solvent (yield % 78; m.p 101–103 °C).

### Refinement

All hydrogen atoms were positioned with idealized geometry (methyl H atoms allowed to rotate but not to tip and were refined with *U*_iso_(H)= 1.2*U*_eq_(C) (1.5 for methyl H atoms using a riding model with C—H = 0.930 for aromatic and and 0.960 for methyl H atoms.

### Crystal data

**Table:**

C_12_H_10_N_2_O_2_S	*F*(000) = 512
*M**_r_* = 246.28	*D*_x_ = 1.397 Mg m^−^^3^
Monoclinic, *P*2_1_/*n*	Mo *K*α radiation, λ = 0.71073 Å
Hall symbol: -P 2yn	Cell parameters from 1442 reflections
*a* = 4.7661 (4) Å	θ = 3.3–27.6°
*b* = 22.8201 (18) Å	µ = 0.26 mm^−^^1^
*c* = 10.7793 (7) Å	*T* = 296 K
β = 92.704 (7)°	Prism, brown
*V* = 1171.08 (15) Å^3^	0.17 × 0.15 × 0.12 mm
*Z* = 4	

### Data collection

**Table:**

Oxford Diffraction SuperNova Eos diffractometer	2211 independent reflections
Radiation source: SuperNova (Mo) X-ray Source	1549 reflections with *I* > 2σ(*I*)
Mirror monochromator	*R*_int_ = 0.064
Detector resolution: 16.0454 pixels mm^-1^	θ_max_ = 26.0°, θ_min_ = 3.3°
ω scans	*h* = −5→5
Absorption correction: multi-scan (*CrysAlis PRO*; Oxford Diffraction, 2009)	*k* = −27→26
*T*_min_ = 0.771, *T*_max_ = 1.000	*l* = −13→8
3997 measured reflections	

### Refinement

**Table:**

Refinement on *F*^2^	Primary atom site location: structure-invariant direct methods
Least-squares matrix: full	Secondary atom site location: difference Fourier map
*R*[*F*^2^ > 2σ(*F*^2^)] = 0.063	Hydrogen site location: inferred from neighbouring sites
*wR*(*F*^2^) = 0.177	H-atom parameters constrained
*S* = 1.05	*w* = 1/[σ^2^(*F*_o_^2^) + (0.0778*P*)^2^] where *P* = (*F*_o_^2^ + 2*F*_c_^2^)/3
2211 reflections	(Δ/σ)_max_ < 0.001
155 parameters	Δρ_max_ = 0.26 e Å^−^^3^
0 restraints	Δρ_min_ = −0.28 e Å^−^^3^

### Special details

**Table:**

Geometry. All e.s.d.'s (except the e.s.d. in the dihedral angle between two l.s. planes) are estimated using the full covariance matrix. The cell e.s.d.'s are taken into account individually in the estimation of e.s.d.'s in distances, angles and torsion angles; correlations between e.s.d.'s in cell parameters are only used when they are defined by crystal symmetry. An approximate (isotropic) treatment of cell e.s.d.'s is used for estimating e.s.d.'s involving l.s. planes.
Refinement. Refinement of *F*^2^ against ALL reflections. The weighted *R*-factor *wR* and goodness of fit *S* are based on *F*^2^, conventional *R*-factors *R* are based on *F*, with *F* set to zero for negative *F*^2^. The threshold expression of *F*^2^ > σ(*F*^2^) is used only for calculating *R*-factors(gt) *etc*. and is not relevant to the choice of reflections for refinement. *R*-factors based on *F*^2^ are statistically about twice as large as those based on *F*, and *R*- factors based on ALL data will be even larger.

### Fractional atomic coordinates and isotropic or equivalent isotropic displacement parameters (Å^2^)

**Table:**

	*x*	*y*	*z*	*U*_iso_*/*U*_eq_	
C1	1.8771 (9)	0.51221 (18)	0.2226 (4)	0.0792 (14)	
H1A	2.0022	0.5083	0.1558	0.119*	
H1B	1.9835	0.5118	0.3005	0.119*	
H1C	1.7765	0.5485	0.2141	0.119*	
C2	1.6709 (7)	0.46177 (17)	0.2185 (4)	0.0576 (11)	
C3	1.5819 (8)	0.43632 (17)	0.1076 (4)	0.0586 (11)	
H3	1.6539	0.4500	0.0343	0.070*	
C4	1.3897 (7)	0.39125 (16)	0.1021 (3)	0.0498 (9)	
H4	1.3321	0.3754	0.0256	0.060*	
C5	1.2814 (7)	0.36934 (15)	0.2103 (3)	0.0409 (8)	
C6	1.3763 (8)	0.39378 (17)	0.3217 (3)	0.0498 (9)	
H6	1.3103	0.3794	0.3956	0.060*	
C7	1.5673 (8)	0.43920 (17)	0.3253 (4)	0.0623 (11)	
H7	1.6273	0.4549	0.4017	0.075*	
C8	0.9566 (7)	0.30543 (15)	0.1167 (3)	0.0436 (8)	
H8	0.9950	0.3238	0.0424	0.052*	
C9	0.7549 (7)	0.25849 (15)	0.1140 (3)	0.0407 (8)	
C10	0.6197 (7)	0.23554 (16)	0.0104 (3)	0.0501 (10)	
H10	0.6498	0.2486	−0.0696	0.060*	
C11	0.4313 (7)	0.19048 (16)	0.0363 (3)	0.0479 (9)	
H11	0.3243	0.1699	−0.0236	0.057*	
C12	0.4260 (7)	0.18089 (14)	0.1598 (3)	0.0379 (8)	
N1	1.0843 (6)	0.32285 (12)	0.2158 (2)	0.0413 (7)	
N2	0.2502 (6)	0.13966 (14)	0.2189 (3)	0.0488 (8)	
O1	0.2643 (6)	0.13733 (12)	0.3329 (2)	0.0663 (8)	
O2	0.0953 (6)	0.10886 (13)	0.1531 (3)	0.0692 (8)	
S1	0.64676 (18)	0.22535 (4)	0.24745 (7)	0.0417 (3)	

### Atomic displacement parameters (Å^2^)

**Table:**

	*U*^11^	*U*^22^	*U*^33^	*U*^12^	*U*^13^	*U*^23^
C1	0.058 (3)	0.047 (2)	0.133 (4)	−0.009 (2)	0.009 (3)	−0.004 (3)
C2	0.036 (2)	0.042 (2)	0.095 (3)	0.0044 (17)	0.008 (2)	0.003 (2)
C3	0.052 (2)	0.050 (2)	0.074 (3)	0.000 (2)	0.010 (2)	0.013 (2)
C4	0.049 (2)	0.051 (2)	0.050 (2)	0.0003 (19)	0.0058 (17)	0.0034 (18)
C5	0.0348 (19)	0.0387 (19)	0.0492 (19)	0.0028 (15)	0.0013 (15)	0.0042 (16)
C6	0.048 (2)	0.052 (2)	0.049 (2)	−0.0071 (19)	−0.0010 (16)	0.0026 (18)
C7	0.055 (2)	0.058 (3)	0.073 (3)	−0.008 (2)	−0.005 (2)	−0.008 (2)
C8	0.042 (2)	0.050 (2)	0.0386 (17)	0.0005 (17)	0.0031 (15)	0.0093 (16)
C9	0.041 (2)	0.044 (2)	0.0360 (17)	0.0019 (16)	−0.0023 (14)	0.0015 (15)
C10	0.051 (2)	0.065 (2)	0.0339 (17)	−0.006 (2)	−0.0021 (16)	0.0042 (17)
C11	0.049 (2)	0.058 (2)	0.0354 (17)	−0.0040 (19)	−0.0052 (15)	−0.0093 (17)
C12	0.0331 (18)	0.0394 (19)	0.0413 (16)	0.0046 (15)	0.0039 (14)	−0.0025 (15)
N1	0.0417 (16)	0.0423 (16)	0.0393 (14)	0.0015 (14)	−0.0032 (12)	0.0033 (13)
N2	0.0507 (19)	0.0464 (19)	0.0493 (17)	0.0056 (16)	0.0041 (15)	0.0033 (15)
O1	0.085 (2)	0.0671 (18)	0.0480 (15)	−0.0064 (16)	0.0113 (14)	0.0092 (14)
O2	0.0684 (19)	0.0685 (18)	0.0704 (17)	−0.0220 (16)	0.0005 (15)	−0.0036 (16)
S1	0.0472 (6)	0.0464 (6)	0.0313 (4)	0.0021 (4)	−0.0015 (4)	−0.0003 (4)

### Geometric parameters (Å, º)

**Table:**

C1—C2	1.513 (5)	C7—H7	0.9300
C1—H1A	0.9600	C8—N1	1.269 (4)
C1—H1B	0.9600	C8—C9	1.439 (5)
C1—H1C	0.9600	C8—H8	0.9300
C2—C7	1.374 (5)	C9—C10	1.366 (4)
C2—C3	1.378 (5)	C9—S1	1.725 (3)
C3—C4	1.377 (5)	C10—C11	1.402 (4)
C3—H3	0.9300	C10—H10	0.9300
C4—C5	1.391 (4)	C11—C12	1.351 (4)
C4—H4	0.9300	C11—H11	0.9300
C5—C6	1.381 (5)	C12—N2	1.430 (4)
C5—N1	1.420 (4)	C12—S1	1.713 (3)
C6—C7	1.379 (5)	N2—O2	1.222 (4)
C6—H6	0.9300	N2—O1	1.228 (4)
			
C2—C1—H1A	109.5	C2—C7—H7	119.3
C2—C1—H1B	109.5	C6—C7—H7	119.3
H1A—C1—H1B	109.5	N1—C8—C9	123.0 (3)
C2—C1—H1C	109.5	N1—C8—H8	118.5
H1A—C1—H1C	109.5	C9—C8—H8	118.5
H1B—C1—H1C	109.5	C10—C9—C8	126.2 (3)
C7—C2—C3	117.4 (4)	C10—C9—S1	111.4 (3)
C7—C2—C1	121.3 (4)	C8—C9—S1	122.4 (2)
C3—C2—C1	121.3 (4)	C9—C10—C11	113.6 (3)
C4—C3—C2	122.0 (4)	C9—C10—H10	123.2
C4—C3—H3	119.0	C11—C10—H10	123.2
C2—C3—H3	119.0	C12—C11—C10	110.9 (3)
C3—C4—C5	120.3 (4)	C12—C11—H11	124.6
C3—C4—H4	119.8	C10—C11—H11	124.6
C5—C4—H4	119.8	C11—C12—N2	125.8 (3)
C6—C5—C4	117.7 (3)	C11—C12—S1	114.1 (3)
C6—C5—N1	117.1 (3)	N2—C12—S1	120.1 (2)
C4—C5—N1	125.2 (3)	C8—N1—C5	119.4 (3)
C7—C6—C5	121.1 (3)	O2—N2—O1	124.0 (3)
C7—C6—H6	119.4	O2—N2—C12	118.1 (3)
C5—C6—H6	119.4	O1—N2—C12	118.0 (3)
C2—C7—C6	121.4 (4)	C12—S1—C9	89.98 (15)
			
C7—C2—C3—C4	2.1 (6)	C9—C10—C11—C12	0.8 (4)
C1—C2—C3—C4	−178.5 (3)	C10—C11—C12—N2	177.6 (3)
C2—C3—C4—C5	−0.9 (6)	C10—C11—C12—S1	−0.2 (4)
C3—C4—C5—C6	−0.9 (5)	C9—C8—N1—C5	179.7 (3)
C3—C4—C5—N1	−179.5 (3)	C6—C5—N1—C8	168.5 (3)
C4—C5—C6—C7	1.4 (5)	C4—C5—N1—C8	−12.8 (5)
N1—C5—C6—C7	−179.9 (3)	C11—C12—N2—O2	2.2 (5)
C3—C2—C7—C6	−1.6 (6)	S1—C12—N2—O2	179.8 (3)
C1—C2—C7—C6	179.1 (4)	C11—C12—N2—O1	−177.9 (3)
C5—C6—C7—C2	−0.2 (6)	S1—C12—N2—O1	−0.2 (4)
N1—C8—C9—C10	−176.6 (3)	C11—C12—S1—C9	−0.3 (3)
N1—C8—C9—S1	5.4 (5)	N2—C12—S1—C9	−178.2 (3)
C8—C9—C10—C11	−179.2 (3)	C10—C9—S1—C12	0.7 (3)
S1—C9—C10—C11	−1.0 (4)	C8—C9—S1—C12	179.0 (3)
